# Modulation of the allosteric and vasoregulatory arms of erythrocytic oxygen transport

**DOI:** 10.3389/fphys.2024.1394650

**Published:** 2024-06-10

**Authors:** Thomas J. Wise, Maura E. Ott, Mahalah S. Joseph, Ian J. Welsby, Cole C. Darrow, Tim J. McMahon

**Affiliations:** ^1^ Duke University School of Medicine, Durham, NC, United States; ^2^ Florida International University School of Medicine, Miami, FL, United States; ^3^ Durham VA Health Care System, Durham, NC, United States

**Keywords:** S-nitrosothiols, ATP, transfusion, sepsis, sickle cell disease, hypoxia, endothelium

## Abstract

Efficient distribution of oxygen (O_2_) to the tissues in mammals depends on the evolved ability of red blood cell (RBC) hemoglobin (Hb) to sense not only O_2_ levels, but metabolic cues such as pH, PCO_2_, and organic phosphates, and then dispense or take up oxygen accordingly. O_2_ delivery is the product of not only oxygen release from RBCs, but also blood flow, which itself is also governed by vasoactive molecular mediators exported by RBCs. These vascular signals, including ATP and S-nitrosothiols (SNOs) are produced and exported as a function of the oxygen and metabolic milieu, and then fine-tune peripheral metabolism through context-sensitive vasoregulation. Emerging and repurposed RBC-oriented therapeutics can modulate either or both of these allosteric and vasoregulatory activities, with a single molecule or other intervention influencing both arms of O_2_ transport in some cases. For example, organic phosphate repletion of stored RBCs boosts the negative allosteric effector 2,3 biphosphoglycerate (BPG) as well as the anti-adhesive molecule ATP. In sickle cell disease, aromatic aldehydes such as voxelotor can disfavor sickling by increasing O_2_ affinity, and in newer generations, these molecules have been coupled to vasoactive nitric oxide (NO)-releasing adducts. Activation of RBC pyruvate kinase also promotes a left shift in oxygen binding by consuming and lowering BPG, while increasing the ATP available for cell health and export on demand. Further translational and clinical investigation of these novel allosteric and/or vasoregulatory approaches to modulating O_2_ transport are expected to yield new insights and improve the ability to correct or compensate for anemia and other O_2_ delivery deficits.

## Background

### Coupling of O_2_ sensing and O_2_ release in red blood cell hemoglobin

Red blood cells (RBCs) operate on several levels to sense and regulate the flux of O_2_ from lung air to tissues and the removal of CO_2_ and other waste products from tissues to lung air. Specifically, RBC hemoglobin (Hb) senses O_2_ tension and adopts an O_2_-binding (in the lungs) or O_2_-releasing (when perfusing the tissues) posture according to host needs and the environment. This tight coupling of O_2_ sensing and demand is fine-tuned and made to fit the metabolic context through relevant allosteric effectors of Hb function such as pH, temperature and CO_2_ tension. Longer-term adjustment of the balance between O_2_ binding and release is accomplished through the generation in RBCs of BPG, which favors O_2_ offloading, for example, in chronic anemia. Additional layers of adaptation recruit increased RBC numbers, for example, via erythropoietin’s coupling of new RBC production to the sensing of hypoxia via hypoxia-inducible factor (HIF) and other regulators.

### Coupling of O_2_ sensing and vascular mediator release by RBCs (and Hb)

**FIGURE 1:**
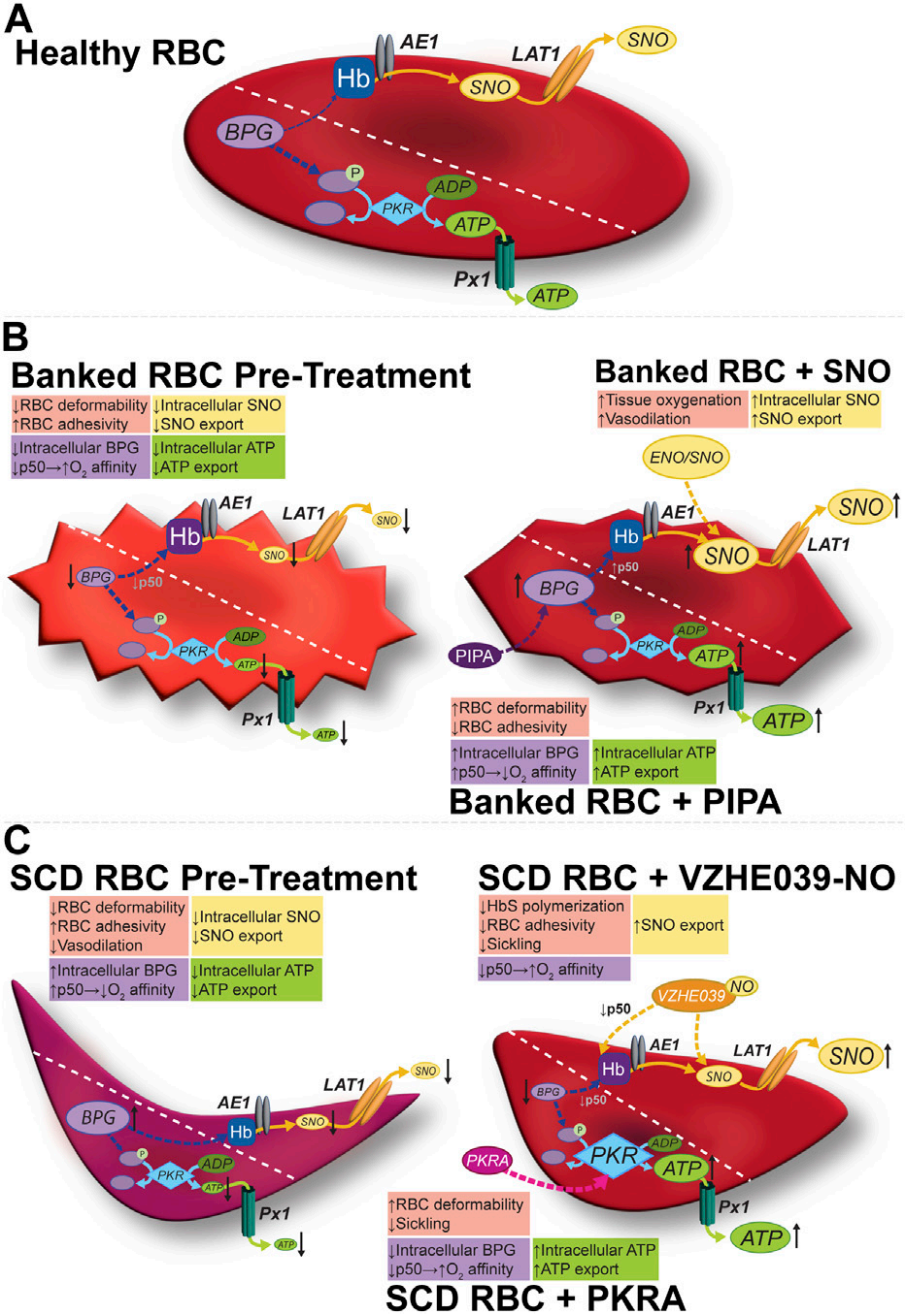
Modulation of the allosteric and vasoregulatory arms of erythrocytic O_2_ transport. **(A)** Glycolysis in healthy RBCs generates the allosteric effector BPG and the ATP vital for cell-intrinsic (e.g., integrity, deformability) and cell-extrinsic (antiadhesive) functions. RBCs export vasoregulatory ATP via Px1 (pannexin 1). SNO is formed on RBC Hb, and can exit via membrane LAT1, possibly after being relayed by AE1. **(B)** In banked RBCs, SNOs and vasoactivity are depressed early (hours) and BPG and ATP are depressed later (weeks). Impaired vasoactivity, heightened O_2_ affinity (lower P_50_), and a proadhesive phenotype (among others) result. The respective mediator and functional changes can be rescued by preserving (e.g., using hypoxic RBC storage) or repleting the precursors (e.g., with PIPA) of organic phosphates or by using a SNO donor such as ENO (ethyl nitrite). **(C)** In sickle RBCs, both SNO and ATP are depressed, in turn depressing vasoactivity. BPG is elevated, shifting O_2_ affinity “rightward (P_50_ is up)” to favor deoxygenation and thus sickling. Activating PKR increases ATP at the expense of BPG (upstream in glycolysis), resulting in resistance to hemolysis and potentially improving ATP export, and disfavoring sickling, respectively. VZHE039-NO stabilizes oxygenated Hb (i.e., lowers P_50_), directly inhibits Hb polymerization (thus preventing sickling two ways), and donates antiadhesive (S)NO. Diagonal dashed lines segregate the BPG/ATP/glycolysis and Hb/SNO/AE1/LAT1 axes for illustration, but these cascades do interact (e.g., BPG and Hb; SNO and GAPDH).

More recently recognized is the ability of the RBC to generate and export vasoregulatory mediators as a function of the metabolic context, a “second arm” of the coupled O_2_ sensing and delivery function of the RBC. For example, an S-nitrosothiol (SNO) group formed on Hb from precursor NO can be relayed to the RBC membrane and subsequently exported to effect hypoxic vasodilation, an allosterically governed fundamental vascular reflex dependent on RBCs (Figure 1). This O_2_-sensitive, allosterically governed blood flow regulation by S-nitrosohemoglobin (SNO-Hb) ensures efficient and well-distributed tissue oxygenation. RBCs also generate and export vasoregulatory and antiadhesive ATP (Figure 1) preferentially in hypoxia, because the docking of deoxygenated Hb at the cytoplasmic domain of the protein band 3 (cdB3) in the RBC membrane allows the cytosolic re-assembly of the glycolytic enzyme complex (which is sequestered on cdB3 when Hb is oxygenated and thus unable to effect glycolysis). Dysregulation of these vasoregulatory activities of the RBC characterize both RBC-intrinsic (e.g., sickle cell disease, malaria, or blood storage) and RBC-extrinsic (e.g., sepsis, diabetes mellitus) diseases. In RBCs, ATP and (S)NO are also critical in cell health, including the maintenance of RBC deformability necessary for its efficient transit through narrow capillaries (Ramdani and Langsley, 2014). In turn, mechanical deformation also triggers ATP release from RBCs and can stimulate endothelial nitric oxide (NO) synthesis, in turn regulating blood flow and the delivery of oxygen (O_2_) to tissues (Ramdani and Langsley, 2014).

Emerging and existing or repurposed RBC therapeutics can modulate the setpoints and gain for O_2_ binding and release, the vasoregulatory mediators harnessed by RBCs for O_2_ delivery, or even both of these two arms of the RBC’s control of O_2_ delivery. In some cases, a single molecule or therapeutic can modulate both RBC arms of O_2_ delivery. Interactions between the former (allosteric effectors) and the latter (vasoregulatory factors) can at least in theory have net additive, neutral, or even inhibitory effects on O_2_ delivery or uptake. Here we review newer therapeutic approaches to restoring RBC function when deficient, as well as mechanistically novel applications of existing molecules. We describe these approaches to modulating RBC function in healthy RBCs and in the context of disease states.

## Red blood cell storage

### RBC storage lesions

#### RBC storage lesions affect RBC quality and transfusion outcomes

While lifesaving for treatment of anemia in selected patients, transfusion of RBCs is neither entirely benign nor frequently beneficial. The process of RBC storage for later transfusion alters the RBC in multiple ways, collectively known as “storage lesions” (Bennett-Guerrero et al., 2007). These storage lesions include changes in RBC structure, composition, and function including decreased BPG, ATP, and SNO levels (Yoshida et al., 2019). These changes contribute to progressive increases in hemolysis, decreasing RBC membrane integrity, and depressed vasoactivity, and may contribute to the well documented lack of benefit (or even harm) of RBC transfusion for many patients with mild to moderate anemia (Hebert et al., 1999; Lacroix et al., 2007; Koch et al., 2008; Wang et al., 2012; Holst et al., 2014).

### Variability in donor RBC metabolism and function

#### Variability in RBC behavior and underlying mediators even among healthy donors

Variation in RBC metabolic activity is not limited to disease states such as PKD, G6PD deficiency, or sickle cell disease, and the inter-donor variability in rates of RBC lysis with cold storage has long suggested metabolic polymorphism. The functional significance of varying RBC ATP was illustrated in a large study of healthy blood donors, along with new genetic insights into its control. Nemkov et al. demonstrated that even after accounting for age, sex and ethnicity, genetic polymorphisms in phosphofructokinase 1 (PFKP), hexokinase 1 (HK1) and the ADP-ribosyl cyclase CD38 accounted for variability in glycolysis in healthy blood. ATP (and other indices of glycolytic activity such as lactate and hypoxanthine) in turn associated significantly with hemolytic propensity *ex vivo* and after RBC transfusion in critically ill patients. *PKLR* (encoding PK in the liver and RBCs) was among the genes for which metabolite quantitative trait loci associated significantly with glycolysis, suggesting a druggable target for potential intervention (Nemkov et al., 2024).

#### RBC transfusion and metabolomics

Large studies of the degree of post-transfusion recovery of RBCs in recipients have revealed substantial variability from donor to donor, and high reproducibility of post-transfusion recovery (PTR) of RBCs from a given donor, consistent with the importance of heritable traits governing this essential measure of transfusion effectiveness. The variability in PTR is clearly linked to variable donor genetics and metabolomes (Nemkov et al., 2024).

#### Altered RBC storage quality secondary to specific donor characteristics

Specific characteristics in donors affect the quality and longevity of stored RBC units derived from their donated blood. Donor obesity, sex, and HbA1c level correlate with stored RBC quality (Hazegh et al., 2021; Tzounakas et al., 2021; Li et al., 2022). The underlying variability has led researchers and clinicians to assert that storage time alone is insufficient to assess quality of stored RBCs (Barshtein et al., 2020). Researchers have explored more accurate methods to non-invasively measure unit hemolysis and quality including sphingomyelinase activity and using spectroscopy (Melzak et al., 2020; Vardaki et al., 2021).

#### Inter-individual variability of RBC O_2_ affinity/P_50_

In general, an individual’s O_2_ carrying capacity can be estimated in part by the hemoglobin (Hb) concentration and the Hb O_2_ saturation (measured, for example, by pulse oximetry). These are important indicators but do not take into account the hemoglobin O_2_ affinity, which plays a key role in O_2_ delivery to tissues. Recent research has shown variation in a patient Hb O_2_ affinity and established that patient-specific normal variants may affect oxygen delivery. While O_2_ dissociation curves are necessary to accurately determine the P_50_, and the hemoglobin O_2_ affinity, estimates in real time of a patient’s (or a donor’s) P_50_ and other indices of O_2_-binding properties from blood gas samples could guide RBC transfusion decision-making more precisely (Lilly et al., 2013; Balcerek et al., 2020).

#### Individualized assessment of RBC qualities and directed use of RBC additives

The study of RBC-omics, a global detailed analysis of all molecules involved in RBC processes, has led to a richer understanding of the complexity of RBC metabolism and signaling. Some omics experts, including D’Alessandro, view the results as indicating that RBCs are not a mere vehicle for oxygen delivery, but also an organ complex interacting bidirectionally with its microenvironment via molecular mediators. This reframing aligns with newly recognized RBC functions (e.g., vasoregulation) and arrives in an era of new technologies in anemia management, such as new additives, evolving medical indications for treatment, and new blood preservation techniques. This new landscape demands the investigation of when, how, and with whom these technologies should be used to mitigate and treat the burden of anemia. RBC omics are helping to answer these questions when paired with new high-throughput devices and machine learning programs (D’Alessandro, 2023).

RBC unit selection for transfusion, D’Alessandro et al. (2023) argue, could be informed by omics and machine learning to personalize decision-making. Current strategies consist of weighing consideration for maximizing inventory usage (first in–first out) with prioritizing fresh units (last in–first out). Detailed study of the units themselves will give physicians more tools to assess the units. Merely using storage duration assumes a direct and proportional correlation between storage duration and storage lesions. This heuristic, while inexpensive and convenient, may not provide the best possible match of patient to blood product. Significant diversity and variation were evident in the genomics of RBC samples, with almost 900,000 polymorphisms found from 13,000 different donors. This variation corresponded with a wide variation in the propensity of blood to hemolyze. In addition to these genomic variations, many medications (acetaminophen, antidepressants, and others) and molecules from donor diets present in donated blood may affect their storage (D’Alessandro et al., 2023).

Some have discussed recent large randomized clinical trials (RCTs) and concluded that adverse events from blood transfusion are not correlated with the storage duration of blood products (Donovan et al., 2022). RBC omics and more precise RBC measurements may offer new insights. RBC gas exchange in the capillaries occurs in seconds in concert with several molecular mediators. Omics may illuminate differences in the patient and blood product that also play a role in the exchange, not controlled for in previous studies. The trials discussed mainly included patients with stable anemia. Excluded, therefore, were populations most affected by the known storage lesions, i.e. those requiring massive transfusions, those with reduced cardiac output, and those at risk for decreased organ perfusion (Donovan et al., 2022). Additionally, “negative” RCTs comparing outcomes after transfusion of RBCs stored “shorter vs longer” would not be expected to reflect the consequences of the set of changes that we and others have demonstrated early in storage (days 0–7) (Bennett-Guerrero et al., 2007).

“Lab-on-a-chip” technology can evaluate in real time individual RBC units for quality and compatibility with recipient patients. This could allow for more specific matching of blood products to patients and would bring blood transfusion into the century of “personalized medicine” (Isiksacan et al., 2023). Nemkov et al. established a high-throughput platform with the ability to inform the development and approval of novel additives to stored RBC. Previously, the process of testing new possible additives has been bottlenecked by a lack of expeditious and efficient evaluation techniques and platforms. Modern platforms could accelerate the process and identify novel candidates for research. Their platform was validated against well-established previous studies (Nemkov et al., 2022).

### Oxygen delivery

#### Disconnect between O_2_ delivery and changes in tissue PO_2_

In hamsters made anemic by isovolemic hemodilution, Cabrales et al. compared the microvascular and systemic effects of transfusion with RBCs of either higher or lower O_2_ affinity than native RBCs, using allosteric effectors electroporated into the cells. In animals receiving high-affinity RBCs, systemic hemodynamics and O_2_ delivery were maintained stable, while tissue PO_2_ decreased (contributing to a steep O_2_ gradient driving O_2_ diffusion to tissues) (Cabrales et al., 2008). In contrast, RBCs with moderately low affinity induced microvascular vasoconstriction, decreased O_2_ delivery and O_2_ extraction, and raised tissue PO_2_. Taken together, these findings point to a disconnect between tissue PO_2_ changes on the one hand, vs changes in O_2_ delivery and O_2_ extraction by tissues on the other, in the face of allosteric modulation in the setting of anemia. The relationship of these observations to physiological benefit or harm has not yet been elucidated.

### Hypoxic blood storage

#### Hypoxic RBC storage

As an alternative to repleting deficient factors lost during blood banking as discussed above, one may modify storage conditions driving these losses to proactively avoid lesion development. One common lesion, oxidative damage, is caused by the buildup of oxidative species secondary to storage in the face of high levels of O_2_. Pittman et al. (2022) discussed how RBCs stored in hypoxemic environments could mitigate this while having no effect on the efficacy of the RBC transfusion. The authors argue that these hypoxically stored RBCs endure exposure to fewer oxidative insults and thus less exposure to the downstream harmful lipid oxidation products following the oxidation of hemoglobin to methemoglobin. These oxidative products may disrupt the normal physiologic functioning of the RBC as well as have inflammatory effects on the patient following transfusion. Yoshida et al. developed and described a method of hypoxic RBC storage, in which the oxygen content in RBC units is lowered before refrigeration and maintained at low levels throughout cold storage. This alternative storage method, now known by the trade name Hemanext, mitigates oxidative stress (driven by abundant O_2_) and thus storage lesion development, and preserves BPG and to some extent ATP, suggesting potential advantages for critically ill and other anemic patients needing RBC transfusion for anemia. Interestingly, while the finding that RBCs stored conventionally become progressively more fully saturated with O_2_ over the typical 6 weeks of storage is generally universal, Yoshida et al. (2017) demonstrated a surprising variability in RBC HbO_2_ saturation levels, even when comparing at the beginning of storage. Accordingly, hypoxic RBC storage could potentially increase the degree of consistency of post-storage (and post-transfusion) RBC functions. More broadly, multiple investigators have highlighted the fact that inter-donor variability in RBC function and metabolic profile is high, and that stored RBC behavior is poorly described in terms of the storage time alone (Dumbill et al., 2023).

#### Advantage of hypoxically stored RBC in hemorrhagic shock

Williams and their team used mouse models of hemorrhagic shock to investigate whether the anaerobic storage of RBC could confer better outcomes than traditionally stored RBC through the reduction of oxidative damage to RBC while not depleting patient oxygen levels. Their model demonstrated that the deoxygenated blood quickly returns to physiologic levels of oxygenation following transfusion and mixing with patient blood in circulation. This, in concert with previous studies validating that anaerobically stored RBCs undergo decreased oxidative damage, advances this storage medium as a promising avenue for further research. In a rat model, resuscitation using hypoxically stored RBCs for transfusion reduce RBC transfusion volumes needed in hemorrhagic shock (Williams et al., 2020).

#### Stored RBC senescence markers modified by hypoxic RBC storage

Bencheikh et al. (2022) demonstrated a protective influence of hypoxic RBC storage (using Hemanext technology) on the appearance of RBC senescence markers (ROS increases, phosphatidylserine (PS) exposure, and calcium entry as assessed by flow cytometry), particularly at 21 and 42 days of otherwise conventional storage. Adhesivity of hypoxically stored RBCs in healthy plasma or in plasma from SCD (at rest or obtained during acute chest syndrome crisis) to thrombospondin (TSP)-1 was significantly attenuated on Day 0, with trends for a beneficial effect of hypoxic storage seen at the longer timepoints (21 and 42 days). The effects of the hypoxic storage of RBCs used to condition subsequent adhesion responses of SCD RBCs to endothelial cells was significant only for 42-day RBCs, and only when a 10% hemolysate was also included in the preconditioning medium. In summary, these findings point to storage-time-dependent beneficial effects of hypoxic RBC storage on indices reflecting senescence changes and adhesivity of sickle RBCs in cell culture models.

#### Metabolic modulation for pRBC oxidative stress due to irradiation

When immunocompromised patients need RBC transfusion, the units are first irradiated to lower the risk of transfusion-associated graft-vs.-host disease. The gamma-(γ-)irradiation used can accelerate the storage-induced adverse changes in RBCs, largely by promoting the storage-associated oxidative changes. The oxidative changes stem in part from the increasing O_2_ levels in the RBC unit. Bardyn et al. (2021) demonstrated that RBC unit storage under conditions of low (and falling) O_2_ and CO_2_ protects against storage-induced deterioration in RBC deformability and the progressive RBC lysis and formation of abnormal spherocytes. The authors consider the possibility that the *in vitro* benefits are secondary to preserved RBC glycolysis and, in fact ATP and BPG levels are better preserved after hypoxic (vs conventional) storage in γ-irradiated RBC units. Consistent with the prediction that preserved BPG stability can promote the ability of stored RBCs to offload O_2_ Rabcuka et al. (2022), Rabcuka et al. demonstrated in Hemanext RBCs superior O_2_ offloading kinetics and a distinct metabolic signature characterized by preserved pyruvate consistent with protection of glycolysis.

### Normoglycemic RBC storage

#### Normoglycemic RBC storage preserves RBC ATP content and export and RBC deformability

Just as functional excess of O_2_ may drive RBC storage lesions, excessive glucose may also be harmful to optimal RBC function. Typical additive solutions contain glucose at >30 mM (five-fold or more over normal blood glucose). Wang et al. (2014) and Liu et al. (2022) demonstrated storing RBCs in normoglycemic conditions may mitigate storage lesions when compared to the current accepted practice of storage under hyperglycemic conditions. Using a 3D-printed transfusion-on-a-chip platform Liu et al. and Spence et al. observed erythrocytes under both storage conditions and in a model of post-transfusion conditions. They found that use of the conventional additive solution AS-1, a hyperglycemic mediums led to a decrease in ATP release and a change in the deformability of the RBC membrane which is reversible upon introduction into their *in vitro* model of transfusion only up to 14 days of storage. Normoglycemic storage medium (“AS-1N”) allowed for RBCs to maintain deformability and release ATP at normal levels for up to 5 weeks, and these “AS-1N” RBCs also responded to stimulation with Zn (zinc) and C-peptide by releasing ATP and deforming maximally. A reduction in storage lesions could decrease the volume of RBC units necessary for resuscitation as well as reduce the adverse effects of transfusion overall. More studies into the storage medium and its role in storage lesions could benefit future anemic patients (Liu et al., 2022). Co-development of technology supporting sustained normoglycemia adds to the translational promise of this improved approach to optimizing RBC function during storage (Soule et al., 2024).

### Rejuvenated stored RBCs (organic phosphate repletion), and roles of other phosphates

#### PIPA (Rejuvesol) and “rejuvenation” of stored RBCs

Depletion of BPG, the negative allosteric effector of O_2_ binding activity of Hb (Figure 1; Table 1) begins to occur during the first week of RBC storage and is effectively complete by the second week. The BPG depletion raises the O_2_ affinity in banked blood, which could limit facile O_2_ delivery to tissues after RBC transfusion and thus be detrimental in certain anemic populations. Secondary additive solutions such as PIPA solutions (containing pyruvate, inosine, phosphate, and adenine; known commercially as Rejuvesol^®^) can be added to RBC units to mitigate this effect as well as related ATP storage lesions and storage-dependent morphological deterioration of the RBC. Such “rejuvenation” was originally performed in the last few days of the typical 6-week storage period but can restore ATP and BPG levels after 2 weeks of storage. In addition to restoring BPG, PIPA treatment of stored RBCs does restore the ability to export ATP upon demand, in turn promoting salutary RBC vasoactivity including the ability to resist adhesion to the endothelium (Kirby et al., 2014).

**TABLE 1:** Changes in erythrocytic allosteric function and vasoregulation in selected diseases or conditions, and effects following some relevant modulatory interventions.

Disease or condition		Intervention		References
**RBC storage lesion**	Effect on RBC allosteric mediators	Allosteric modulator		
		Name and mechanism	Effect	
	↓ATP export, late ↓ ATP content; ↓BPG, ↑Oxygen affinity; ↓S1P; ↓SNO	PIPA—Rejuvesol: RBC rejuvenation using a solution of pyruvate, inosine, phosphate and adenine	↑ATP content and export; ↑BPG, ↓Oxygen affinity; ↑RBC deformability, adhesivity, and oxygen delivery	Kirby et al. (2014), Srinivasan et at. (2018), Rabcuka et al. (2022)
		Hypoxic RBC storage—Hemanext: Decreases oxidative stress inherent to conventional storage	↑ATP content and export; ↑BPG, ↓Oxygen affinity; ↓Post-transfusion inflammation; reduces RBC volume needed to resuscitate after hemorrhage	Yoshida et al. (2017), Nazeman et al. (2022), Pittman et al. (2022), Williams et al. (2020)
		Normoglycemic storage: Prevents lesions secondary to conventional storage at more than 5× normal glucose levels	↑ATP export; ↑RBC deformability	Wang et al. (2014), Liu et al. (2022), Soule et al. (2024)
	Effect on RBC function and vasoregulation	Vasoregulatory modulator		
		Name and mechanism	Effect	
	↓RBC deformability; ↑RBC adhesivity; ↓Vasoactivity; ↓Survival; ↓Oxygen delivery	S1P supplementation: Promotes RBC glycolysis by mediating the binding of hemoglobin to the N-terminus of the RBC membrane anion transporter Band 3	↑ATP content and export; ↑BPG, ↓Oxygen affinity; ↑RBC deformability, adhesivity, and oxygen delivery; ↓ NADPH	Hay et al. (2023)
		Ethyl nitrite (ENO): SNO donor repletes pathologically deficient level in recipient	↑RBC SNO, ↓RBC adhesivity, ↑RBC deformability, ↑Hypoxia-induced vasodilation	Riccio et al. (2015), Reynolds et al. (2007)
Sickle cell disease (SCD)	Effect on RBC allosteric mediators	Allosteric modulator		
		Name and mechanism	Effect	
	↓ATP content and export; ↑BPG, ↓Oxygen affinity	PIPA—Rejuvesol	↓BPG, ↑Oxygen Affinity, ↓RBC transfusion dependence	Lopez Domowicz et al. (2020)
		Polymerization inhibitor—Voxeletor: Increases Hb oxygen affinity, favoring oxyHb state	↓BPG, ↑Oxygen Affinity, ↓RBC sickling and polymerization; ↑Hb levels; ↓Hemolysis	Suhail (2024), Howard et al. (2021), Shah et al. (2022)
		Polymerization inhibitor/NO donor—VZHE-039-NO: Increases Hb oxygen affinity AS WELL AS directly interrupts polymerization and donates vasoactive NO	↓BPG, ↑Oxygen affinity, ↓RBC sickling and polymerization; ↑Hb levels; ↓Hemolysis; ↓RBC adhesivity	Huang et al., 2022; Abdulmalik et al.,
	Effect on RBC function and vasoregulation	Vasoregulatory modulator		
		Name and mechanism	Effect	
	Decreased deformability; increased adhesivity; decreased vasoactivity; increased RBC polymerization; ↓SNO	RBC-specific pyruvate kinase activator (PKRA)—Mitapivat, Etavopivat: Activation of pyruvate kinase R increases production of ATP through RBC glycolysis; lowers BPG	↑RBC ATP content and export; ↓BPG, ↑Oxygen affinity; ↑RBC Deformability, ↓RBC Sickling; ↓Hemolysis	Quezado et al. (2022)
		ENO	↑RBC SNO, ↓RBC adhesivity, ↑RBC deformability, ↑Hypoxia-induced vasodilation	Reynolds et al. (2023), Pawloski et al. (2005)
Malaria	Effect on RBC allosteric mediators			
	↑ATP export → ↑Parasitemia			
	Effect on RBC function and vasoregulation	Vasoregulatory modulator		
		Name & mechanism	Effect	
	↓RBC deformability, ↑Hemolysis	Purinergic P2Y receptor inhibitor —KN-62, Ip5I: Inhibition of Pxn 1 channels; decreases RBC ATP export and extracellular ATP	↓ATP export → ↓Parasitemia	Tanneur et al. (2006); Levano-Garcia et al. (2010); Alvarez et al. (2014)
		E-NTPDase inhibitor: Inhibition of this P. falciparum specific ectonucleotidase decreases the hydrolysis of ATP to AMP, thus decreasing RBC cAMP levels	↓RBC cAMP, ↓ATP export, ↑RBC deformability → ↓Parasitemia	Borges-Pereira et al. (2017), Paul et al. (2019)
Pyruvate kinase deficiency (PKD)	Effect on RBC allosteric mediators			
	↓ATP content and export; ↑BPG, ↓Oxygen affinity			
	Effect on RBC function and vasoregulation	Vasoregulatory modulator		
		Name & mechanism	Effect	
	Hemolysis	PKRA	↑PK enzymatic activity, ↑RBC Glycolysis, ↑ATP content and export	Ayi et al. (2008); Al-Samkari et al. (2022)
β-Thalassemia	Effect on RBC allosteric mediators			
	↓ATP content and export; ↑BPG, ↓Oxygen affinity			
	Effect on RBC function and vasoregulation	Vasoregulatory modulator		
		Name and mechanism	Effect	
	Hemolysis	PKRA	↑ATP export; ↑Erythropoiesis; ↓Oxidative stress; ↑Mitochondrial function	Al-Samkari et al. (2022); Matte et al. (2021)
Pulmonary arterial hypertension	Effect on RBC function and vasoregulation	Vasoregulatory modulator		
		Name and mechanism	Effect	
	↓SNO; Decreased vasoactivity	ENO	↑RBC SNO, ↑RBC vasoactivity, Improved pulmonary hemodynamics	McMahon et al. (2005), Moya et al. (2001), Moya et al. (2002)

In practice, however, and despite FDA approval, PIPA is seldom used outside of its application in the cryopreservation of rare blood types. Gehrke et al. and Evans et al. tested the effects of an approach that makes PIPA incubation (aka “rejuvenation”) more practical: the addition of PIPA at Day 3 of cold storage. This avoids the conventional (but cumbersome) one-hour, 37°C incubation of the RBC unit with PIPA (conditions linked to its FDA-approved clinical use) (Gehrke et al., 2018; Gehrke et al., 2019). Cold PIPA incubation, like conventional PIPA use, increased ATP and BPG levels (Figure 1, middle panels) to just above the upper limit of normal and mitigated the storage-induced increase in O_2_ affinity, without engendering additional RBC lysis or vulnerability to lysis of the RBCs in a benchtop model of a cardiopulmonary bypass circuit. This approach could improve post-storage RBC function not only via maintenance of the P_50_ at near-normal values, but also via the increased erythrocytic ATP, which is necessary for enzymes functioning to defend RBC integrity and for blood flow-regulating vasoactivity that fine-tunes O_2_ delivery. Indeed, cold “rejuvenation/PIPA treatment” attenuated storage-induced declines in deformability and the progressive increases in mechanical fragility and RBC lysis (Evans et al., 2020). Among other potential downstream mechanisms of the benefits of preserving these critical organic phosphates, they may inhibit fatty acid desaturases, in turn limiting fatty acid accumulation (Thomas et al., 2021). When the ability of Rejuvesol to restore metabolites in RBCs stored over an extended period was studied, it was found to be effective in restoring BPG and ATP levels in RBCs stored in multiple mediums for up to 120 days. While the stored cells responded to “rejuvenation” less over time, when rejuvenated their BPG and ATP levels exceeded those in fresh blood for 72- and 96-hours post-treatment, respectively (Meyer et al., 2011; Smethurst et al., 2019). Notably, following transfusion, BPG is gradually regenerated in the transfused RBCs, with levels ultimately matching those of the recipient by 72 h (Valeri and Hirsch, 1969; Heaton et al., 1989).

#### Transfusion with “rejuvenated” blood (PIPA RBCs) could increase tissue O_2_ delivery

As stated previously, RBC storage results in an increase in hemoglobin oxygen affinity (decreased P_50_), in part due to depletion of BPG. This increase in oxygen affinity may have particularly deleterious effects in anemic patients undergoing cardiac surgery or massive hemorrhage. These populations are particularly susceptible due to limited cardiac reserve and risk of decreased organ perfusion. Srinivasan et al. asked the question “Could one unit of low oxygen affinity blood offer the same benefits of two units of standard blood?” They created and utilized an *in vitro* model of transfusion to assess this question. Their simulated model demonstrated that ‘rejuvenated’ units could incrementally decrease hemoglobin oxygen affinity (increase P_50_) following transfusion with 1, 2 and 3 units compared to standard stored RBC units. While standard RBC transfusion increased oxygen delivery, rejuvenated units were calculated to increase oxygen utilization in the tissues based on calculations of arteriovenous oxygen content difference. For patients with robust reserve of cardiac output, standard RBC transfusion may not confer negative outcomes secondary to its increased hemoglobin oxygen affinity, but in patients with reduced cardiac reserve, treatment of perioperative anemia with rejuvenated RBC units may promote improved outcomes (Srinivasan et al., 2018). This assertion is also supported by evidence of better metabolic resuscitation in an animal hemorrhage model using blood with preserved BPG and ATP versus standard storage (Williams et al., 2020)*, as discussed later in the section on hypoxic blood storage methods. While clinical studies comparing outcomes after rejuvenated versus standard RBC treatment, are lacking it has been demonstrated that P_50_ does decrease *in vivo* after large volume transfusions, and this decrease can be ameliorated by transfusing rejuvenated/PIPA treated RBCs. In a pilot study in sickle cell disease (SCD) patients undergoing red blood cell exchange (RCE) transfusion therapy, standard RCE was compared with RCE using the last 4 RBC units treated with PIPA. The findings indicated that PIPA-treated RCE maintained RBC oxygen affinity (consistent with the preservation of BPG), and more generally identified favorable or neutral effects on key metabolic and vascular biomarkers in chronically transfused SCD patients (Lopez Domowicz et al., 2020).

#### PIPA, BPG, and O_2_ offloading kinetics *in vitro*

Efficient and responsive offloading of O_2_ is an essential function of RBCs. Although the O_2_-binding characteristics of Hb and RBCs are widely understood through O_2_ equilibrium curves, the kinetics of O_2_ fluxes is also critical. Using a microfluidic chamber designed to rapidly switch between oxygenated and hypoxic perfusate and fluorescent probes reading hemoglobin O_2_ saturation, Rabcuka et al. (2022) demonstrated in banked RBCs that single-cell O_2_ desaturation kinetics in hypoxia are superior after hypoxic storage (Hemanext^TM^ storage system) or after mid-storage PIPA loading (“rejuvenation”) as compared to those after conventional RBC storage. The benefits of hypoxic RBC storage persisted until about 35 days of storage. Metabolomic signatures common to the benefits of rejuvenation and hypoxic RBC storage were identified, as were signatures unique to each. In contrast, only a few proteins were significantly protected from oxidation by hypoxic storage, and no distinguishing lipidomic signature was identified.

#### Diffusion-limited state and the effects of PIPA/BPG in perfused kidneys

The clinical significance of changes in the kinetics of O_2_ binding and release, or in the position and shape of the O_2_ dissociation curve in general, has been debated. In banked RBCs, for example, the leftward shift driven in part by depletion of BPG has been viewed as only a minor concern. One argument holds that PO_2_ in blood will equilibrate with that of the tissues during the time it takes the RBC to traverse the capillary; higher-affinity RBCs may take longer (the argument goes) but offloading ultimately does take place during transit. This condition in O_2_ delivery is sometimes referred to as a “perfusion-limited” state. Dumbill et al. (2023) recently published elegant new findings challenging this contention. In explanted human kidneys considered for transplantation, they demonstrated that perfusion with PIPA-treated RBC transfusates, which returns the P_50_ and O_2_-offloading time constant toward that of fresh RBCs, resulted in 60% higher renal cortical PO_2_ as compared to perfusion with control stored RBCs from the same donor. These findings support a “diffusion-limited” model of O_2_ delivery, in which the O_2_-offloading kinetic properties of the perfusing RBCs play an important role in O_2_ transfer. The diffusion-limited model may be particularly relevant in organs with higher O_2_ needs, including the brain, skeletal muscle, and the heart; when regional blood flow is elevated (shortening RBC transit time), and when anemia is present.

#### Should microcirculatory indices be investigated to inform decision-making for RBC transfusion?

Microcirculatory functions that govern tissue perfusion are logical indices to guide medical decision-making and act as therapeutic targets, but progress has been limited by a paucity of evidence of their incremental value beyond standard parameters (macrohemodynamic indices like blood pressure, cardiac output, and pulse oximetry) and by uncertainty over the relevance of microcirculatory data from accessible circulatory beds (e.g., the sublingual microvasculature). In critically ill adults, Van Manen et al. (2020) identified significant differences in the RBC-transfusion-induced change in microcirculatory indices (proportion of perfused vessels and microvascular flow index) in patients with greater illness severity as compared to those with moderately severe illness, as defined by SOFA (sequential organ failure assessment) scores. The results suggest that the addition of a microcirculatory index in decision-making over about RBC transfusion is worthy of study. Given the emergence of clinically accessible modulators of both arms (O_2_ kinetics and vasoregulation) of the control of O_2_ delivery by RBCs, it is also tempting to speculate that integration of a microcirculatory endpoint in decision algorithms could identify critically ill patient endotypes (subsets of patients) who may benefit from transfusion with modified units of RBCs that are (for example) poised to offload O_2_ more efficiently, poised to vasodilate more readily, or both (or neither). Alternatively, differential (or mutual) regulation of these RBC O_2_-delivery functions could be influenced independent of the need for RBC transfusion by systemic (oral) administration of agents in these therapeutic classes.

#### Metabolic effects of S1P in the RBC

Multiple approaches to augmenting organic phosphates (ATP and/or BPG particularly) in either native or transfused (stored) RBCs have been demonstrated. In contrast to the PIPA approach which depends upon boosting substrate/precursors, agents that promote glycolysis enzymatically or via competition (described below) are also effective but may have different advantage/disadvantage profiles. Sphingosine-1-phosphate (S1P) promotes RBC glycolysis by mediating the binding of hemoglobin to the N-terminus (cytoplasmic tail) of the RBC membrane anion transporter Band 3 (also termed anion exchanger 1 (AE1)). This drives glycolysis by freeing up the complex of glycolytic enzymes that otherwise remain inactivated by assembling on the cytoplasmic domain of Band 3 (cdB3). Because RBC storage leads to loss of S1P, supplementing S1P is logical. Hay et al. demonstrated the ability of exogenous S1P (“dosed” so as to restore pre-storage levels) boosted RBC ATP and BPG levels. However, this came at the expense of generation of NADPH, a reductant generated via the pentose phosphate pathway (PPP), whose activity is blunted when glycolysis accelerates due to substrate competition. While the immediate result may be disappointing, there is reason to reconsider the S1P approach, for example, in combination with provision of additional substrate and/or under conditions (such as *in vivo*) where RBCs are cycling normally between oxygenated (promoting PPP activity and NADPH generation) and deoxygenated (promoting glycolysis and thus ATP and BPG synthesis) states (D’Alessandro et al., 2023; Donovan et al., 2022; Isiksacan et al., 2023; Nemkov et al., 2022; Nielsen et al., 2017).

## Red blood cells in disease states

### PKD and thalassemia

#### Pyruvate kinase, ATP, and hemolytic anemias

The sole pathway for the generation of ATP in red blood cells is glycolysis, with pyruvate kinase generating ATP from ADP late in glycolysis. Persons with PK deficiency (PKD) are susceptible to hemolytic anemia. PKD patients are relatively protected from infection with malaria, an effect that may have driven the high frequency of the *PKLR* genetic variants in the sub-Saharan African population (Ayi et al., 2008). This paradoxical protective effect is reminiscent of the protective effect of HbS against malarial infection. Conversely, when heterozygous HbS (“sickle trait”) and PKD coincide, an SCD phenotype emerges. In two independent cohorts of child and adult patients with HbSS or HbSβo (beta-thalassemia) SCD, certain *PKLR* variants were demonstrated to associate with the frequency of acute pain episodes requiring hospitalization (Wang et al., 2022). These findings underscore a modulatory role for RBC PK (PKR) in SCD outcomes and symptoms and are supportive of investigation of the use of PKR activators in reducing the frequency of such acute pain episodes and other pathophysiology, especially in individuals with such *PKLR* variants (Wang et al., 2022). PKD is characterized by chronic hemolytic anemia and iron overload. The benefits of pyruvate kinase activation are several: improvements are seen in erythropoiesis and iron homeostasis. Recent clinical reports have indicated that the PKR activators etavopivat and mitapivat have beneficial effects in other etiologies of anemia beyond PKD, including beta-thalassemia.

#### PKR activation in PKD and thalassemia

The pyruvate kinase (PK) activators AG-348 (mitapivat, by Agios Pharmaceuticals) and FT-4202 (known as etavopivat, originally by Forma now Novo Nordisk), have shown early promise in addressing various hereditary hemolytic anemias, in PK deficiency (PKD) and beyond. In a study investigating PKD, a rare hereditary condition affecting red blood cell (RBC) glycolytic metabolism, AG-348 effectively increased PK enzymatic activity and stability in PK-deficient RBCs, apparently restoring glycolytic pathway activity (Rab et al., 2021). In phase III clinical trials for PK deficiency, mitapivat was shown to be safe and efficacious, with results suggesting its potential as a disease-modifying therapy for hereditary hemolytic anemias (Al-Samkari and van Beers, 2021). Mitapivat also showed promise in treating β-thalassemia-related anemia by improving erythropoiesis, reducing oxidative stress, and enhancing mitochondrial function (Matte et al., 2021). Additionally, mitapivat demonstrates potential beyond these disorders, showing efficacy in hereditary spherocytosis according to preclinical studies (Matte et al., 2021). Clinical trials focusing on PK deficiency patients reveal mitapivat’s capacity to improve markers of ineffective erythropoiesis and iron homeostasis, offering a potential reduction in iron overload. With convenient oral administration and a safety profile comparable to placebo in adults with PK deficiency, mitapivat and etavopivat have emerged as promising new therapeutic options for various hereditary hemolytic anemias, including those lacking currently approved drug therapies (Schroeder et al., 2022; Shrestha et al., 2021). The results suggest that PKRAs have the potential to be effective and disease-modifying therapies for PK deficiency, offering early and robust Hb responses and the normalization of Hb levels in a significant proportion of patients.

### Sickle cell disease

#### Allosteric modulation using voxelotor in SCD

The hallmark transformation to sickle (crescent)-shaped cells in sickle cell disease (SCD) takes place upon SCD RBC deoxygenation as HbS gains the ability polymerize when in its deoxygenated, but not in the oxygenated, state. Polymerization leads to the formation of long, insoluble fibers that stretch and distort the RBC, ultimately forcing the sickle shape. The polymerization of sickled HbS leads to vaso-occlusive crises (VOCs) in patients, and the number of sickled RBCs has been shown to rise one to three days before the clinical presentation of VOC (Table 1). Polymerized sickled RBCs have decreased deformability, obstruct the microvasculature, and promote end-organ damage, as well as intense pain for the patient. VOCs drive much of the morbidity and mortality in SCD with hospitalization necessary for 95% of VOC presentations (Darbari et al., 2020; Suhail, 2024). Metcalf et al. (2017) investigated the use of positive allosteric RBC modulators (that can increase the oxygen affinity of hemoglobin) to increase the proportion of HbS in the oxy-HbS state. The aim was to assess whether stabilizing HbS in the oxygenated state could prevent polymerization. They found success with a compound, then called GBT440, which through reversible and covalent binding to hemoglobin, stabilized the oxygenated state and limited polymerization. Advantageously, it was also found to be orally bioavailable and partitioned to RBC at an RBC/plasma ratio of 150, allowing for low systemic concentrations while still having a therapeutic effect. A large phase III randomized trial, the Hemoglobin Oxygen Affinity Modulation to Inhibit HbS Polymerization (HOPE) Trial, showed this molecule, now known as voxelotor, to be safe and effective and led to its FDA approval for treatment of sickle cell disease (Vichinsky et al., 2019; Howard et al., 2021). In these trials, voxelotor increased hemoglobin levels, decreased the incidence of anemia, and decreased hemolysis. However, treatment was not associated with any change in frequency of VOCs. Since its approval, voxelotor has been associated with lower transfusion requirements, fewer prescribed opiates, and an increase in mean Hb as compared to standard therapy (Shah et al., 2022). Overall, voxelotor has been a proof of concept for the therapeutic power and clinical impact of the altering Hb O_2_ affinity.

With multiple new interventions including voxelotor and PKR activators (acting to suppress BPG) such as etavopivat, there is now a need to compare therapies. Moody et al. (2024) created a quantitative model allowing comparison of each intervention with an effective dose of hydroxyurea (induction of endogenous HbF to 30%) and modeling how the two medications might work together (which can also reduce the required dosing of each agent). This clinical tool, and others like it, could, if validated, guide providers in their choice of therapy and dosage for treatment of SCD. Investigating other potential benefits of voxelotor in SCD patients, Mendelson et al. (2024) used a mouse model to show that of voxelotor could replace the several months of RBC transfusion currently required prior to gene therapy for SCD. New assays will allow the future identification of novel anti-sickling compounds (Nakagawa et al., 2022). These new roles for voxelotor, as well as the discovery of novel compounds, could increase the overall availability and use of disease-modifying therapy (DMT) for SCD, as rates of DMT use remain low even with the introduction of newer therapies (Newman et al., 2023).

#### Voxelotor in acute lung injury and hypoxemia

The ability to manipulate the oxygen affinity of hemoglobin may also be leveraged in situations of hypoxia outside of SCD. There is a theoretical advantage to left-shifting the oxygen dissociation curve, and thus increasing the oxygen affinity for Hb, in hypoxemia. This intervention allows for increased oxygen binding at a given PO_2_, which could increase the uptake of oxygen in the lungs in critically ill patients including those with acute lung injury. While voxelotor is only FDA-approved at this time for use in sickle cell disease, recent studies explored its usage in other settings. Vlahakis et al. (2019) showed the promise of this therapy in idiopathic pulmonary fibrosis (IPF) patients, showing that voxelotor decreased exercise-induced hypoxemia in a small cohort of IPF patients. Stewart et al. (2020) showed voxelotor could increase arterial oxygen saturation in healthy patients during hypoxia and submaximal exercise. With the goal of reducing hypoxemia by means other than via supplemental oxygen delivery (which can itself be toxic), improving ventilation/perfusion (V/Q) mismatch, and limiting RBC transfusion, centers have begun to trial voxelotor with patients receiving intensive care. Two such cases in critically ill patients experiencing hypoxemia were treated with voxelotor at Duke University by one of the authors. While clinical benefit cannot be established by case reports, no adverse effects from the trial were noted and the tolerability of the therapy was demonstrated (Al-Qudsi et al., 2023). More robust testing with large RCTs is necessary to further assess the efficacies and roles of these interventions.

Voxelotor has been successful in increasing Hb levels in patients but relying on this singular metric as a heuristic for oxygen supply to tissues could lead to misunderstanding and even patient harm. The oxygen carrying capacity of Hb as well as its ability to offload oxygen are also important factors in transferring oxygen from the lungs to tissues. Increasing the oxygen affinity of hemoglobin allows for increased onloading of oxygen in the lungs, but, conversely, may disfavor or decrease unloading of oxygen to the tissues. This fact has led to concern around the possibility that these DMTs that increase O_2_ affinity of Hb could have the negative effect of decreased tissue O_2_ delivery. Longer-term follow-up of participants in the HOPE trial has shown no end-organ perfusion related damage secondary to voxelotor treatment, although these studies might lack the power needed to find such changes (Howard et al., 2021). Alternatively, the lack of net harm (via impaired O_2_ offloading in the tissues), if confirmed, could reflect compensatorily increased activity of local microcirculatory regulators including ATP and SNO.

#### O_2_ affinity in SCD and the rationale(s) for PKR activation

PKR activation is rational in SCD not only because boosting RBC ATP may be beneficial, but also by lowering BPG levels resulting in a favorable change in O_2_ affinity (Figure 1; Table 1). O_2_ affinity and dissociation behavior in SCD blood differ from that of healthy blood in several respects. BPG levels are increased in SCD, but the degree of change varies (Table 1; Figure 1). Overall, the resulting “rightward shift” in the O_2_ binding curve is small, and depressed O_2_ saturation of HbS arises more from increases in CO-Hb (carbonmonoxyHb; CO is generated endogenously as a byproduct of heme turnover following hemolysis) and in oxidized metHb (Needleman et al., 1999). Variable compensatory upregulation of production of fetal Hb moves O_2_ affinity in the opposite direction (higher). Finally, once polymerized (as during SCD crisis), functional HbS affinity is lower. Pulse oximetric readings of Hb O_2_ saturation may be misleading for two major reasons: first, there is a now well-recognized algorithmic bias in pulse oximetry based SpO_2_ readings owing to subject skin color (pigment), with major implications in patients of African descent (Wong et al., 2021). Secondly, the SpO_2_ values are skewed by the presence of CO-Hb and metHb as noted.

The investigational erythrocyte pyruvate kinase (PKR) activator etavopivat was also studied in clinical trials (Xu et al., 2022) focused on sickle cell disease (SCD) patients (Figure 1). These trials aimed to identify the maximum dose with an acceptable safety profile. Cohorts of patients with SCD treated were with varying doses of etavopivat for 2 weeks; improvements were seen in various markers and the drug was well-tolerated according to safety profiles (Forsyth et al., 2022). In an open-label study of patients treated with etavopivat for 12 weeks, similarly reassuring results were demonstrated in terms of safety, along with improved markers of anemia and hemolysis (Saraf et al., 2024). These findings suggest that etavopivat could be an effective treatment for SCD patients, potentially reducing the risk of vaso-occlusive crises and end-organ damage. Etavopivat may hold promise for the treatment of sickle cell disease and other hemoglobin disorders by targeting some of the underlying pathophysiology. Interestingly, the benefits in SCD may reflect not only the increased RBC ATP levels, but also decreased BPG, as described below.

#### PKR activation in a mouse model of SCD

In sickle cell disease, RBC sickling and its downstream consequences depend on HbS (sickle hemoglobin) deoxygenation. In the SCD RBC, deoxygenation is favored due to elevated levels of BPG. Treatments that lower the BPG concentration and thereby raise O_2_ affinity (lower P_50_) are therefore predicted to have therapeutic benefit (Table 1). Indeed, indirect (e.g., hydroxyurea, which stimulates production of high-O_2_-affinity fetal hemoglobin, HbF) and direct (voxelotor) approaches to increase O_2_ affinity have demonstrated benefits in SCD. RBCs from persons with SCD contain and export lower amounts of vasoregulatory ATP, which may contribute to the dysregulation of the microcirculation in this disease, as manifested acutely by vasoocclusion and chronically by increased susceptibility to ischemic strokes. Activation of PKR in SCD is therefore therapeutically attractive for at least two effects: it can raise intra-RBC ATP, which is important for both numerous cell-intrinsic functions such as preserving cell integrity and minimizing hemolysis and for extrinsic RBC actions such as vasoregulatory effects of ATP. Additionally of benefit in SCD is that PKR activation can lower BPG. In both human SCD and the Berkeley SCD mouse model, BPG levels are elevated, and ATP is depressed as compared to controls (Jensen et al., 1973; Zhang et al., 2011; Shrestha et al., 2021; Forsyth et al., 2022; Schroeder et al., 2022). In contrast, the Townes SCD mouse model is characterized by upregulated PKR protein, elevated ATP, and decreased BPG (Quezado et al., 2022). Nevertheless, the PKR activator mitapivat increased RBC ATP values further while having no effect on BPG levels in Townes mice. In parallel, the PKRA induced favorable changes in RBC mitochondrial retention, RBC oxidative tone, and leukocytosis but no significant attenuation of sickling threshold. These findings suggest that increases in RBC ATP alone may be beneficial in SCD, even when baseline ATP values are near normal. In sickle cell disease, mitapivat’s ability to increase ATP levels and reduce complications in a mouse model underscores its potential therapeutic effects, although the differences between mouse models and actual human SCD are acknowledged (Al-Samkari and van Beers, 2021; Matte et al., 2021).

#### Dual- and triple-action drugs for SCD: allosteric modulation, anti-polymerization, and vasoregulatory

Therapeutic approaches to anti-sickling have focused on both measures to disfavor facile deoxygenation and methods to inhibit polymerization. Hydroxyurea is an established therapy in SCD that induces the production of fetal hemoglobin (HbF). HbF has higher O_2_ affinity than adult Hb (accounting for the ability of the fetus to extract O_2_ from maternal blood), and its presence alongside HbS can prevent deoxyHbS from achieving the critical concentrations necessary for polymerization. More recently, voxelotor (Oxbryta) gained FDA approval, and this aromatic aldehyde raises O_2_ affinity, disfavoring the deoxygenation-dependent polymerization process. Next-generation aromatic aldehydes such as VZHE-039 (Abdulmalik et al., 2020), developed and synthesized by Dr. Martin Safo et al., not only raise O_2_ affinity (thus preventing HbS polymerization through an “O_2_-dependent” anti-sickling mechanism), but also have direct anti-polymerization action through direct interactions with the alpha subunits of HbS. Safo et al. went further and incorporated into VZHE-039 a nitric oxide (NO)-donor moiety by synthesizing the nitrate ester derivative of VZHE-039, VZHE-039-NO (Figure 1) (Huang et al., 2022). This molecule retains both the O_2_-dependent (allosteric) and O_2_-independent (direct) anti-polymerization properties while introducing antiadhesive effects on the treated SCD RBCs by delivering the NO group (Huang et al., 2022). We have also demonstrated antiadhesive actions of NO/SNO repletion using simple NO donors in SS RBCs (McMahon et al., 2019).

#### RBC ATP and malaria: the dark side of RBC-derived ATP

Following parasitic infection with *P. falciparum*, ATP release has been found to contribute to parasitic growth via various mechanisms. ATP release is stimulated by surges in intracellular cyclic adenosine monophosphate (cAMP) concentrations in response to hypoxia or mechanical stress (Table 1). (Sluyter, 2015) Binding of ATP to purinergic receptors, specifically P2Y receptors, on the cell membrane induces the opening of “new permeability pathways (NPP),” channels for osmolytes and anions (Tanneur et al., 2006) whose entry facilitate growth of the parasite as nutrients such as carbohydrates and amino acids can be imported intracellularly and metabolic waste can be removed (Tanneur et al., 2006; Ramdani and Langsley, 2014). Additionally, binding of RBC P2Y receptors by the released ATP has been linked to an upregulation of cAMP production, associated with decreased deformability of malaria-infected cells due to phosphorylated cytoskeletal proteins, establishing positive feedback for the further release of ATP (Ramdani and Langsley, 2014; Sluyter, 2015). The reduced deformability can contribute to pathophysiology by rendering these RBCs more susceptible to the lysis seen in malarial disease.

ATP is normally far more abundant in the RBC than in the plasma. Since increased extracellular ATP content has been associated with increased rates of parasitemia (Alvarez et al., 2014), preventing release of ATP may be a new approach to limiting parasitic growth and infection. ATP is primarily released through pannexin 1 (Px1), a membrane channel or pore that facilitates the passive export of ATP; therefore, blocking the release of ATP could minimize extracellular ATP content (Alvarez et al., 2014). Widely used anti-malaria drugs, such as mefloquine, block the Px1 channel and their success in combating malaria infection appears to be tied to prevention of ATP release (Dahl et al., 2013) in addition to their direct anti-parasite actions. With the rise of parasitic resistance to currently-available anti-malarial drugs (Borges-Pereira et al., 2017), another possible avenue for malaria therapeutics is the use of inhibitors of purinergic P2Y receptors, such as KN-62 and Ip5I, which have been associated *in vitro* with reduced levels of parasitemia in human RBCs (Tanneur et al., 2006; Levano-Garcia et al., 2010). Purinergic receptors have additionally been linked to opening Px1 channels, consequently increasing extracellular ATP (Locovei et al., 2006), so inhibition of P2Y receptors could potentially also limit ATP release by disrupting the feedback loop. Further research regarding selective inhibitors of purinergic receptors could be advantageous for preventing the opening of NPPs and changes in the deformability (fragility) of infected RBCs. Another potential target of therapy are the ectonucleotidases, extracellular enzymes typically located on the surface of RBCs and other cells that can hydrolyze extracellular ATP molecules into AMP, which can then be converted to adenosine, a molecule that signals to increase of cAMP levels within RBCs (Borges-Pereira et al., 2017; Paul et al., 2019). As mentioned, increased intracellular cAMP content stimulates the further release of ATP and additionally increases the rigidity of RBCs (Paul et al., 2019). The genome of *P. falciparum* contains a specific ectonucleoside E-NTPDase whose activity is heightened with higher levels of extracellular ATP (Alvarez et al., 2014). Inhibition of E-NTPDase hinders the development of infected RBCs, emphasizing the link between ectonucleotidases and parasitic growth (Borges-Pereira et al., 2017).

### RBC SNO depletion and cardiovascular/cardiopulmonary disease

#### RBC vasoactivity and SNO

The binding and release of NO by RBC Hb are allosterically controlled by the oxygenation and deoxygenation-induced toggling between the R (relaxed) and T tense) conformations of Hb. Upon oxygenation, an SNO adduct forms from precursor NO at the reactive and highly conserved β93 Cys thiol residue of Hb. Conversely, and in keeping with basic thermodynamics, the SNO moiety is released from Hb upon the allosteric transition to the deoxygenated, T structure. The released SNO can exit the RBC, unlike precursor NO whose affinity for the heme groups in RBC Hb so great that escape is exceedingly rare. SNO-Hb displays higher O_2_ affinity and a leftward shift in the O_2_ dissociation curve relative to unmodified Hb. This elevated O_2_ affinity acts to disfavor profligate SNO release but has no meaningful impact on aggregate blood O_2_ affinity because only about 1 per 1000 Hb molecules carries a SNO group, and the ODC shift is not huge. Stated otherwise, blood (RBC) Hb is densely concentrated (millimolar), but RBC Hb-bound SNO is in low abundance (∼1 μM). Nevertheless, given the high vasoregulatory potency of SNOs, nanomolar fluxes of this vascular signal resulting from the release of only a small fraction of RBC Hb-derived SNO are sufficient to effect blood flow-regulating vasodilation. We recently identified the type 1 system L amino acid transporter (LAT1) as the conduit responsible for SNO export by RBCs (Figure 1) and its import by endothelial cells. LAT1 inhibitors diminish the extracellular accumulation of SNOs that is typical when RBCs are deoxygenated, and in a mouse deficient in endothelial LAT1 (LAT1^ECKD^), cellular uptake of CSNO is impaired (Dosier et al., 2017). The broad vasoregulatory purview of RBC-derived SNOs is underscored by the observation that when LAT1^ECKO^ mice are transfused, recipient RBCs are sequestered in the lungs and blood oxygenation is depressed. These findings are reminiscent of the impaired oxygenation typical of patients transfused with stored RBCs, which are depleted of SNOs.

Some investigators questioned the importance of SNO-Hb in vasoregulation. In particular, RBCs from a mouse model bearing human hemoglobin in which the β93 Cys residue was mutated to Ala (alanine) were reported to function normally. But this mouse was also engineered to express gamma hemoglobin [a component of fetal hemoglobin (HbF)], which we have shown is also reversibly S-nitrosylated (Riccio et al., 2016); this rescue may account for the lack of phenotype in this mouse. By contrast, Zhang and coworkers demonstrated that even the persistence (or presence by knock-in) of SNO-susceptible fetal hemoglobin does not fully compensate for the mutation of the critical Cys normally present at residue 93 of the beta-globin subunit of hemoglobin (Zhang et al., 2015). In mice where β93 Cys is mutated to Ala, peripheral blood flow is depressed at baseline and declines during hypoxia, rather than increasing, which is the classic adaptive peripheral vascular response. Accordingly, tissue oxygenation is lower at baseline in the C93A mice than in transgenic controls expressing non-mutated human Hb and declines further during hypoxia. In humanized mouse models of myocardial infarction and heart failure (Zhang et al., 2016), mutation of Hb at the relevant Cysβ93 residue rendering it incapable of forming and transferring the SNO group led to greater cardiac injury and mortality. Also underscoring the essential nature of this RBC activity was that in the mutant mice, coronary vessel collateralization was demonstrated (but did not suffice to prevent injury and mortality) (Zhang et al., 2016).

The regional nature of the hypoxic vasodilatory reflex is exemplified in reactive hyperemia, in which blood flow to an organ (a leg, for example) rebounds higher than baseline flow following the release of a briefly enforced arterial occlusion (e.g., by tourniquet). Reynolds and coworkers found RH responses, and the associated post-reperfusion rebound in tissue oxygenation, to be deficient in C93A mutant mice as compared to mice expressing wild-type (C93) human Hb. In humans, the time needed for tissue reoxygenation upon reactive hyperemic responses correlated inversely with both SNO-Hb absolute values and with the ratio of SNO-Hb to total Hb-bound NO. In patients with peripheral arterial disease, tissue reoxygenation was slowed and SNO-Hb values were depressed. Taken together these findings indicate a role for SNO-Hb in the metabolite-driven (and O_2_-sensitive) hyperemic response to reperfusion, a clinically relevant adaptive response involving hypoxic vasodilation and RBCs (Reynolds et al., 2023).

In an elegant test of the role of RBCs, NO and SNO in human hypoxic vasodilation, Hoiland et al. (2023) demonstrated that changes in cerebral blood flow in response to hypoxia were associated with increases in the transcerebral [arterial-to-jugular venous (A-V)] SNO gradient, but not associated with a cerebral A-V nitrite gradient. Cerebral hypoxic vasodilation was augmented during hemodilution in both lowlanders and in polycythemic native Andeans living at high altitude (4300 m), underscoring the apparent role of vasoregulatory mediators downstream of the exquisite O_2_ sensor hemoglobin. Taken together, these findings support the assertion that the hypoxia-driven release of SNOs (perhaps ultimately formed from precursor NO by endothelial-type NO synthase) from RBCs contributes critically to the characteristically O_2_-sensitive vasoregulation typical of the brain.

#### Modulation of RBC-dependent vasoregulation by SNO donors

Deficient RBC-based SNO-dependent vasoactivity contribute to pathology in disease states including SCD, pulmonary arterial hypertension, and ischemic cardiovascular disease (McMahon et al., 2005; Pawloski et al., 2005; Zhang et al., 2015; Sonveaux et al., 2007). RBC transfusion for anemia only benefits a subset of patients: those with moderate or severe anemia (Hb < 7 gm/dL in several randomized studies) (Hebert et al., 1999; Lacroix et al., 2007), and the early, deficient vasoregulatory capacity of stored blood secondary to depletion of SNO and ATP appears contributory. One possible exception to the general lack of benefit of more aggressive RBC transfusion is the patient with acute myocardial infarction, with the recently reported MINT trial showing a strong trend in outcomes (Carson et al., 2023) interpreted by some (Bloch and Tobian, 2023) as supporting a more liberal transfusion strategy in these patients and, by extension, a recognition that one size does not fit all in RBC transfusion decision-making. In the heart, a high O_2_ utilization downstream of flow-limiting stenosis and/or thrombosis may contribute to benefits of RBC transfusion outweighing its potential harms.

#### Restoration of RBC vasoactivity

Decreased vasoactivity due to diminished RBC export of SNO can be restored to more physiologic levels by either direct exposure of the RBCs or by administration to patients of SNO precursors. In banked human RBCs deficient in SNO, exposure to NO donors under the appropriate conditions is sufficient to regenerate SNO using the ability of Hb to form SNO from NO (Riccio et al., 2015). In stored RBCs this SNO restoration improves RBC deformability and attenuates RBC adhesivity (Riccio et al., 2015). RBCs exposed to the SNO donor ethyl nitrite (ENO) regain their ability to effect hypoxic vasodilation (Reynolds et al., 2007). In adults PAH patients breathing ENO, RBC SNO and RBC vasoactivity are restored, with parallel improvements in pulmonary hemodynamics (phenocopying that seen in animals) (McMahon et al., 2005; Moya et al., 2001). Newborns with persistent pulmonary hypertension see similar pulmonary hemodynamic benefits (Moya et al., 2002).
